# Supplementary material for: Comparing Patient Characteristics, Clinical Outcomes, and Biomarkers of Severe Asthma Patients in Taiwan
Source: Biomedicines. 2021 Jul 1;9(7):764. doi: 10.3390/biomedicines9070764 (PMC8301447; doi:10.3390/biomedicines9070764)
Supplement: Supplementary file 1 [file biomedicines-09-00764-s001.zip › biomedicines-1244828-supplementary.pdf]

Supplemental table 1 Treatment

The biomarker group with EOS count ≥ 300 and FeNo measure ≥ 20 had more patients treated with ICS/LABA/LAMA, LTA, omalizumab and prednisolone, while other biomarker groups with other combination of EOS count and FeNo measure had more patients using ICS alone, Theophyline, and antihistamine.

BIOMARKER GROUP	All patient	ICS alone		ICS/LABA		ICS/LAMA		ICS/LABA/LAMA		LTA		Theophylline		Antihistamine		Omalizumab		Pred	
	N	N	%	N	%	N	%	N	%	N	%	N	%	N	%	N	%	N	%
**CRITERIA SET #1**																			
EOS <300 & FeNO < 20	26	1	3.85	20	76.92	1	3.85	4	15.38	8	30.77	18	69.23	3	11.54	1	3.85	1	3.85
EOS ≥300 & FeNO < 20	6	1	16.67	3	50.00	0	0.00	1	16.67	3	50.00	5	83.33	1	16.67	2	33.33	0	0.00
EOS <300 & FeNO ≥ 20	62	1	1.61	42	67.74	0	0.00	19	30.65	27	43.55	40	64.52	3	4.84	4	6.45	4	6.45
EOS ≥300 & FeNO ≥ 20	33	0	0.00	20	60.61	2	6.06	11	33.33	17	51.52	17	51.52	1	3.03	3	9.09	3	9.09
missing	5																		
**CRITERIA SET #2**																			
EOS ≥300 & FeNO ≥ 20	33	0	0.00	20	60.61	2	6.06	11	33.33	17	51.52	17	51.52	1	3.03	3	9.09	3	9.09
Others	94	3	3.19	65	69.15	1	1.06	24	25.53	38	40.43	63	67.02	7	7.45	7	7.45	5	5.32
missing	5																		
**CRITERIA SET #3**																			
EOS <150	50	1	2.00	38	76.00	0	0.00	11	22.00	17	34.00	33	66.00	2	4.00	2	4.00	3	6.00
EOS ≥150	81	2	2.47	51	62.96	3	3.70	24	29.63	39	48.15	47	58.02	6	7.41	8	9.88	5	6.17
missing	1																		
**CRITERIA SET #4**																			
EOS <200	71	1	1.41	51	71.83	1	1.41	18	25.35	28	39.44	44	61.97	3	4.23	2	2.82	3	4.23
EOS ≥ 200	60	2	3.33	38	63.33	2	3.33	17	28.33	28	46.67	36	60.00	5	8.33	8	13.33	5	8.33
missing	1																		
**CRITERIA SET #5**																			
EOS <250	78	1	1.28	56	71.79	1	1.28	20	25.64	30	38.46	49	62.82	3	3.85	3	3.85	3	3.85
EOS ≥250	53	2	3.77	33	62.26	2	3.77	15	28.30	26	49.06	31	58.49	5	9.43	7	13.21	5	9.43

missing	1																		
**CRITERIA SET #6**																			
EOS <300	90	2	2.22	64	71.11	1	1.11	23	25.56	35	38.89	58	64.44	6	6.67	5	5.56	5	5.56
EOS ≥300	41	1	2.44	25	60.98	2	4.88	12	29.27	21	51.22	22	53.66	2	4.88	5	12.20	3	7.32
missing	1																		
**CRITERIA SET #7**																			
FeNO <20	32	2	6.25	23	71.88	1	3.13	5	15.63	11	34.38	23	71.88	4	12.50	3	9.38	1	3.13
FeNO ≥20	96	1	1.04	63	65.63	2	2.08	30	31.25	44	45.83	57	59.38	4	4.17	7	7.29	8	8.33
missing	4																		
**CRITERIA SET #8**																			
FeNO <25	57	2	3.51	39	68.42	2	3.51	13	22.81	23	40.35	42	73.68	4	7.02	4	7.02	3	5.26
FeNO ≥25	71	1	1.41	47	66.20	1	1.41	22	30.99	32	45.07	38	53.52	4	5.63	6	8.45	6	8.45
missing	4																		
**CRITERIA SET #9**																			
FeNO <30	73	2	2.74	51	69.86	2	2.74	17	23.29	32	43.84	51	69.86	5	6.85	5	6.85	4	5.48
FeNO ≥30	55	1	1.82	35	63.64	1	1.82	18	32.73	23	41.82	29	52.73	3	5.45	5	9.09	5	9.09
missing	4																		
**CRITERIA SET #10**																			
FeNO <35	81	3	3.70	55	67.90	2	2.47	20	24.69	36	44.44	55	67.90	5	6.17	6	7.41	4	4.94
FeNO ≥35	47	0	0.00	31	65.96	1	2.13	15	31.91	19	40.43	25	53.19	3	6.38	4	8.51	5	10.64
missing	4																		
**CRITERIA SET #11**																			
FeNO <40	93	3	3.23	61	65.59	3	3.23	25	26.88	41	44.09	60	64.52	5	5.38	6	6.45	4	4.30
FeNO ≥40	35	0	0.00	25	71.43	0	0.00	10	28.57	14	40.00	20	57.14	3	8.57	4	11.43	5	14.29
missing	4																		
**CRITERIA SET #12**																			
EOS <150 & FeNO < 25	22	0	0.00	17	77.27	0	0.00	5	22.73	8	36.36	17	77.27	2	9.09	1	4.55	1	4.55

EOS ≥150 & FeNO < 25	35	2	5.71	22	62.86	2	5.71	8	22.86	15	42.86	25	71.43	2	5.71	3	8.57	2	5.71
EOS <150 & FeNO ≥ 25	27	1	3.70	20	74.07	0	0.00	6	22.22	9	33.33	16	59.26	0	0.00	1	3.70	2	7.41
EOS ≥150 & FeNO ≥ 25	43	0	0.00	26	49.06	1	1.89	16	30.19	23	43.40	22	41.51	4	7.55	5	9.43	3	5.66
Missing	5																		
